# The impact of rejection sensitivity on suicidal ideation among college students: the role of social connectedness and negative affect

**DOI:** 10.1038/s41598-026-58201-x

**Published:** 2026-07-03

**Authors:** Weiman Yan, Tao Xu, Siqi Ma, Chen Hong, Yu Wang, Wei Liu

**Affiliations:** 1 School of Psychology, Shanghai Normal University, Shanghai, China; 2 Psychology Section, Second Sanatorium of Air Force Healthcare Center for Special Services, Hangzhou, China; 3 Psychological Research Center, Xinjiang Taineng Psychological Counseling Institution, Xinjiang, China

**Keywords:** Rejection sensitivity, Social connectedness, Negative affect, Suicidal ideation, College students, Health care, Psychology, Psychology, Risk factors

## Abstract

This study aimed to explore the relationship between rejection sensitivity and suicidal ideation among college students and to examine the chain-mediated role of social connectedness and negative affect in this association. Using a cross-sectional survey design, 1152 college students (43.75% female, 56.25% male) were recruited through stratified cluster convenience sampling and completed measures assessing rejection sensitivity, social connectedness, negative affect, and suicidal ideation. Bivariate correlations were examined, and mediation analyses were conducted to test whether social connectedness and negative affect independently and sequentially mediated the association between rejection sensitivity and suicidal ideation. Rejection sensitivity was positively associated with negative affect and suicidal ideation, and negatively associated with social connectedness. Social connectedness and negative affect each independently mediated the relationship between rejection sensitivity and suicidal ideation, and they also functioned as chain mediators in this relationship. Overall, rejection sensitivity was both directly and indirectly associated with suicidal ideation through the chain-mediated effects of social connectedness and negative affect.

## Introduction

According to statistics from the World Health Organization (WHO), approximately 700,000 people die by suicide globally each year, which is equivalent to one suicide every 40 s. Suicide is the third leading cause of death among young people aged 15–29 worldwide^1^. Suicidal ideation is defined as thinking about, considering, or planning suicide^2^, which is regarded as a critical precursor to suicidal behavior^3^. Relative to other youth subpopulations, Chinese college students have been consistently identified as a high-risk population for suicidal problems^4^. A meta-analysis revealed that the prevalence of suicidal ideation among Chinese college students is approximately 10%^5^. Given this alarming prevalence, identifying the specific risk factors for suicidal ideation in this population is an urgent research priority.

One key set of risk factors stems from the distinctive sociocultural and environmental characteristics of Chinese campuses. China is a collectivist country, and Chinese college students grow up in a cultural environment that emphasizes collectivism^6^. Upon entering college, they are required to live in group settings^7^. For Chinese college students, unlike their Western counterparts, the individual is understood as inherently belonging to the collective, making the establishment of interpersonal connections a crucial developmental task^6,8^. Importantly, this culturally rooted emphasis on interpersonal relationships coexists with escalating academic and occupational pressures, which together shape the stressful social ecology of contemporary Chinese college campuses^9^.

Specifically, over the past decade, the number of college graduates in China has surged due to the higher education expansion policy, while the growth rate of job opportunities has failed to keep pace, which has been accompanied by intensified competition in the labor market and severe “involution” on campus^10,11^. The intense academic competition among students is associated with both interpersonal alienation and higher levels of negative affect, such as depression and anxiety^12,13^. Beyond these universal competitive pressures, a culturally specific factor, namely face concern, further exacerbates psychological distress. Face concern refers to the social reputation and interpersonal dignity that Chinese individuals highly value. This social reputation relies on recognition and positive evaluation from surrounding social members^14^. Because of this cultural trait, students may be reluctant to admit psychological difficulties, which in turn amplifies their distress. Specifically, disclosing emotional distress or seeking professional psychological help is often stigmatized as a sign of personal incompetence, leading many students to avoid seeking social support or counseling^15,16^. Consequently, even when support resources are available, they remain underutilized, leaving psychological problems unaddressed.

### Rejection sensitivity and suicidal ideation

As a stable trait closely tied to interpersonal evaluation and exclusion stress, rejection sensitivity has emerged as a pivotal risk factor for adolescent psychological maladjustment. Rejection sensitivity is defined as the individual tendency to anxiously expect, readily perceive, and overreact to rejection^17,18^. In collectivist cultural contexts that prioritize interpersonal harmony, this trait becomes especially consequential. Chinese college students are inherently more attentive to others’ social evaluations and more sensitive to interpersonal relationship quality^19,20^. Their strong motivation to sustain harmonious interpersonal relationships also induces intense fear of social rejection and exclusion^21^. Because social rejection directly threatens both relational harmony and face, students high in rejection sensitivity are particularly vulnerable to perceiving rejection where it may not exist. Individuals with high rejection sensitivity are more prone to overreact when faced with real or perceived rejection, which is associated with psychological adjustment problems such as depression and social avoidance^18,22,23^. Accordingly, rejection sensitivity is widely regarded as a crucial stable vulnerability factor for negative psychological outcomes^22,24^.

Furthermore, consistent with this vulnerability perspective, rejection sensitivity is also a stable cognitive-affective disposition that shapes responses to social cues, with its most pronounced manifestations and detrimental effects emerging specifically in intimate relationships^18^. This heightened sensitivity often co-occurs with contradictory interpersonal behaviors, such as hostility, avoidance, or excessive appeasement, during social interactions, which may in turn undermine the development of stable social connections with others^18,25^. According to the Rejection Sensitivity model^26^ individuals who have experienced repeated rejection are prone to forming defensive expectations of rejection. These expectations lead them to become hypervigilant to potential rejection cues, making them inclined to misinterpret ambiguous or neutral behaviors as intentional exclusion. This perceived rejection immediately triggers emotional reactions such as negative affect, which in turn induce a series of maladaptive behaviors, ultimately contributing to the occurrence of actual rejection and forming a complete cycle.

The college period is a key developmental stage where rejection sensitivity exerts prominent adverse effects on mental health. First, college students are in a critical transitional stage of interpersonal development with strong intrinsic needs for social interaction and intimate relationship establishment^27^. Second, the transition from a single-family environment to a complex collective campus environment brings unprecedented interpersonal challenges and uncertainty^28^. This transition can easily activate the rejection expectations characteristic of high rejection sensitivity^26^. Third, in addition to traditional offline peer interactions, the prevalence of online social platforms has further exacerbated the psychological consequences of rejection sensitivity. As the most active group of social media users in China, college students are frequently exposed to online social comparison and instant social feedback mechanisms such as likes, comments, and private messages^29–31^. This online social context makes students overly focused on others’ public evaluations. A lack of positive online feedback or ambiguous social responses is easily interpreted as social exclusion and rejection, further aggravating their interpersonal anxiety^31,32^. Thus, under the dual pressures of offline and online interactions, highly rejection-sensitive college students face severe interpersonal adaptation difficulties.

To understand the relationship between these difficulties and suicidal ideation, the Interpersonal Theory of Suicide provides a useful theoretical framework. This theory posits that the co-occurrence of two core negative interpersonal states, namely thwarted belongingness and perceived burdensomeness, induces and sustains suicidal ideation when individuals perceive these states as persistent and unchangeable^33,34^. Specifically, thwarted belongingness refers to the painful psychological state caused by unmet interpersonal connection needs, while perceived burdensomeness reflects the negative cognition of being a burden to others arising from unmet social competence needs^35^. Because rejection sensitivity directly threatens one’s sense of social belonging, it is particularly likely to increase both thwarted belongingness and perceived burdensomeness. Thus, rejection sensitivity functions as a distal dispositional vulnerability factor rather than a direct cause of suicidal ideation. It elevates individuals’ susceptibility to thwarted belongingness and perceived burdensomeness, thereby indirectly increasing suicide risk^33^.

Before presenting the specific hypotheses, it is worth clarifying how rejection sensitivity differs from conceptually related constructs. Its defining feature is a specific focus on the anxious anticipation of exclusion, which is particularly relevant for understanding deficits in social connectedness and subsequent negative affect. Interpersonal sensitivity is defined as encompassing both (a) a tendency to be excessively worried about others’ negative evaluations, and (b) a tendency to excessively react to such negative evaluations^36^. While rejection sensitivity shares a concern with negative interpersonal feedback, its defining feature is a specific focus on social rejection, specifically the anxious expectation and overreaction to perceived exclusion or rejection, rather than a broader sensitivity to any form of negative evaluation. Privileged self, in contrast, is defined as a tendency to pursue one’s own pleasure at the expense of maintaining harmony with others^37^. This conceptual distinction confirms that rejection sensitivity uniquely captures fear of interpersonal exclusion. However, compared to those with low levels of rejection sensitivity, individuals with high levels of rejection sensitivity tend to exhibit higher levels of suicidal ideation^38^. Based on this, the research Hypothesis 1 (H1): there is a significant positive correlation between rejection sensitivity and suicidal ideation among college students.

### Mediating effect of social connectedness

Having established rejection sensitivity as a dispositional vulnerability factor, we next consider the mechanisms through which it may relate to suicidal ideation^39^. Social connectedness is defined as the subjective awareness of being in close relationship with the social world, encompassing a sense of belonging, attachment, and interpersonal relationships^40^. The relationship between social contact quantity and social connectedness is complex. While some research indicates that larger interaction networks are associated with lower intimacy^41,42^, other studies have shown that having more friends is negatively associated with loneliness^43^. In contrast, interaction quality demonstrates a more consistent positive predictive effect: higher quality social interactions are associated with a stronger sense of social connectedness^44^.

Critically, social connectedness is a robust protective factor against suicide. Each additional social connection is associated with a 39% reduction in suicide risk^45^. Specifically, forming and maintaining social connections play a crucial role in enhancing belongingness^46^. Social connection offers the experience of acceptance and inclusion, fulfilling people’s basic need for belonging^47,48^. Moreover, strong social connectedness can enhance an individual’s belongingness, mitigate anxiety and stress in social interactions, and provide opportunities to support others, which, in turn, can weaken perceived burdensomeness and reduce suicidal ideation^49,50^. Notably, in addition to psychological resources, strong social connectedness can also provide material resources, such as financial aid or help with practical tasks, to help individuals cope with adversity^51^. Individuals who can borrow $500 when needed have a 72% lower risk of engaging in suicidal behavior compared to those who cannot^52^. Research indicates that higher levels of social connectedness are associated with a lower risk of developing suicidal ideation, and this finding holds consistently across contexts such as school, family, and peer relationships^53^.

The link between rejection sensitivity and diminished social connectedness is well established. Individuals with high rejection sensitivity are chronically worried about rejection and tend to limit interpersonal interaction and emotional expression with others, instead adopting a defensive, emotionally distant interpersonal style^34,54^. Empirical evidence shows that individuals with higher rejection sensitivity engage in significantly less physical contact during social interactions than those with lower rejection sensitivity^55^. In the short term, this self-protective strategy may effectively help them avoid immediate rejection^56,57^. However, over time, persistent avoidance prevents the disconfirmation of rejection expectations, and this pattern is associated with chronic social withdrawal^57^. This pattern of withdrawal not only hinders the development of social skills but also paradoxically increases the likelihood of actual peer rejection^23,58^. Through this cyclical process, individuals with high rejection sensitivity ultimately fail to establish and maintain the social connections essential for a sense of belongingness. Therefore, we propose Hypothesis 2 (H2): Social connectedness mediates the relationship between rejection sensitivity and suicidal ideation among Chinese college students.

### Mediating effect of negative affect

Beyond social connectedness, rejection sensitivity may also be associated with suicidal ideation through negative affect. Negative affect is a general dimension of subjective distress and unpleasurable engagement that subsumes a variety of aversive mood states, including anger, contempt, disgust, guilt, fear, and nervousness^59^. In social interactions, individuals sensitive to social rejection readily develop dysfunctional expectations that others will reject them, which can trigger intense negative affect, such as stress, anxiety, anger, rumination, and helplessness^60^. Furthermore, the tendency of individuals with high rejection sensitivity to be hypervigilant and overreactive to rejection from others activates the defensive motivational system^61^, making them more susceptible to negative affect such as depression and anxiety^18,25^. A meta-analytic study also showed that rejection sensitivity is positively correlated with various forms of negative affect, and this association remains stable in longitudinal studies^62^.

Notably, in addition to directly generating negative affect through exaggerated reactions to perceived rejection, rejection sensitivity may further impair individuals’ ability to regulate such negative affect, namely emotion dysregulation^23,63^. In other words, negative affect represents the emotional states arising from rejection sensitivity related overreactions, whereas emotion dysregulation reflects failures in the regulatory processes required to manage and alleviate these aversive emotional states^64,65^. Existing research also indicates that rejection sensitivity is indirectly associated with increased negative affect through emotion dysregulation^23^.

In research on suicidal ideation, negative affect is one of the most widely examined risk factors. According to the escape theory of suicide, suicide may result from standards that are perceived as too high, or from experiencing particularly adverse circumstances^66^. Chinese college students hold high expectations for interpersonal interactions; therefore, once they experience actual rejection, they perceive themselves as failing to meet their expected standards^67,68^. The discrepancy between reality and expectations triggers the negative affect of disappointment, and the magnitude of this discrepancy serves as a decisive factor for suicide^66^. Additionally, when individuals perceive that they have failed to meet certain important standards, they may also experience negative affect such as depression, guilt, and anxiety.

When intense negative affect cannot be effectively regulated, individuals may adopt cognitive deconstruction (constricted temporal focus, concrete thinking, immediate or proximal goals, cognitive rigidity, and rejection of meaning) to escape aversive self-awareness and emotional distress^66^. Therefore, cognitive deconstruction should not be considered emotion dysregulation itself, but rather a maladaptive escape response that may emerge when individuals are unable to effectively regulate overwhelming negative affect^69^. The consequences of cognitive deconstruction, such as disinhibition, are closely linked to suicide^66^. For Chinese college students, the cultural pressure to maintain interpersonal harmony and avoid losing face may lower the threshold at which negative affect becomes unbearable^14^. Empirical studies have also provided substantial support for this. Previous cross-sectional research has shown a direct positive link between negative affect and suicidal ideation^70,71^. Longitudinal studies have similarly found that increases in negative affect are associated with an increased risk of suicidal ideation^72^. Furthermore, network analysis studies have found that negative affect, such as anxiety, panic, depression, and guilt, is significantly associated with suicidal ideation^73–75^. Given this, this study proposes Hypothesis 3 (H3): Negative affect mediates the relationship between rejection sensitivity and suicidal ideation among college students.

### The chain mediation effect of social connectedness and negative affect

While we have separately hypothesized mediating roles for social connectedness and negative affect, a more integrated theoretical perspective suggests that these two mechanisms may operate sequentially. Drawing on Self-Determination Theory, belongingness constitutes a fundamental psychological need^76^. The Rejection Sensitivity model posits that individuals high in rejection sensitivity adopt defensive social withdrawal as a self-protective strategy, which, as argued above, undermines social connectedness, the subjective sense of being in close relationship with one’s social world^18,40^. According to the Interpersonal Theory of Suicide, this deficit in social connectedness represents a state of thwarted belongingness, a key proximal risk factor for suicidal ideation^34^.

Critically, the frustration of belongingness needs does not merely increase suicide risk directly; it also generates considerable negative affect^77,78^. Individuals with low social connectedness may have fewer emotional regulatory resources that supportive relationships provide, which may be related to greater vulnerability to negative affective states such as anxiety and depression^48^. Consistent with Escape Theory^66^, such negative affect becomes an aversive state from which individuals seek to escape, and suicidal ideation may emerge as a cognitive manifestation of this escape motivation. Thus, rather than operating as independent parallel pathways, social connectedness and negative affect may form a sequential chain: rejection sensitivity is associated with lower social connectedness (reflecting thwarted belongingness), which in turn is associated with higher negative affect. Based on this, this study proposes Hypothesis 4 (H4): Social connectedness and negative affect exert a chain-mediated effect on the relationship between rejection sensitivity and suicidal ideation.

**Fig. 1:**
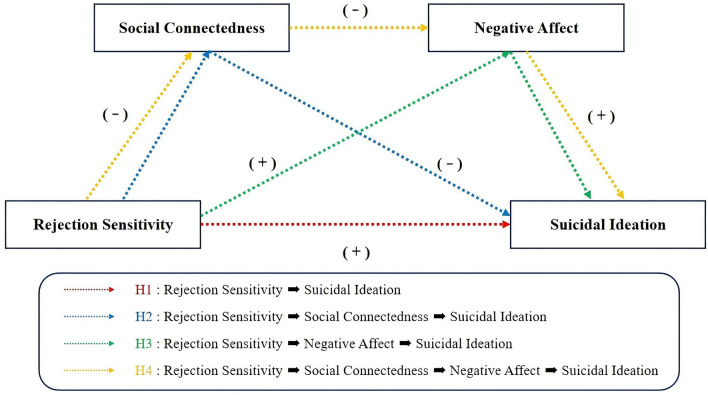
The conceptual model.

As a result, the research hypothesis model is constructed as shown in Fig. 1.

## Method

### Participants and procedure

This cross-sectional study was conducted in China. Data were collected using convenience sampling methods via online questionnaires. Participants were invited to complete a series of self-report questionnaires through Wenjuanxing, a reliable and secure Chinese online survey platform^9^. Inclusion criteria were an age of at least 18 years, self-reporting good health with no history of neurological or psychiatric disorders, and providing informed consent. After being fully informed about the study procedures, all participants electronically signed an informed consent form. The study was conducted anonymously to ensure the authenticity of the collected data. The study respected participants’ autonomy, and withdrawal was permitted at any stage of the research process.

In addition to the four main variables reported in this manuscript (rejection sensitivity, social connectedness, negative affect, and suicidal ideation), we also collected basic demographic information including age, gender, grade level, and whether the participant was an only child. No other psychological variables were collected. Participants were recruited through online announcements posted on college bulletin boards and WeChat groups. The study was described as a “survey on college students’ mental health and social experiences.” Before participating, all participants read an electronic informed consent form that clearly explained the purpose of the study, the voluntary nature of participation, the anonymity of responses, and the right to withdraw at any time without penalty. Only those who provided informed consent proceeded to complete the questionnaires.

To ensure the rigor of data preprocessing, we implemented a multi-step screening procedure to define and exclude invalid responses. Specifically, invalid responses were identified based on the following three criteria: (1) logical inconsistencies, determined by failing to pass any of the three embedded attention-check items (e.g., Please select “strongly agree”); (2) excessively short completion time, defined as completing the entire questionnaire in less than 100 seconds^79^, which was empirically determined as insufficient for thoughtful completion based on the pilot test; and (3) elevated dissimulation scores, as indicated by a score of 4 or higher on the SIOSS dissimulation factor, which suggests a tendency to conceal true responses. After excluding invalid responses, a total of 1,152 valid datasets were obtained, with a mean age of 21.38 years (SD = 2.02), and an age range of 18 to 26 years, yielding a validity rate of 96%. The final sample comprised 648 males (56.25%) and 504 females (43.75%). Our study received approval from the Ethics Committee of Shanghai Normal University.

### Measures

#### Negative affect

Negative affect was assessed by the Negative Affect subscale of the Positive and Negative Affect Schedule (PANAS) developed by Watson et al.^59^. Participants were asked to indicate the extent to which they had experienced each negative affect during the past 1–2 weeks. The Negative Affect subscale comprises 10 items (e.g., distress, shame, irritability), and each item is scored on a 5-point Likert-type scale (“1” representing very slightly or not at all and “5” representing extremely). The final score was determined by calculating the mean score of all items, with a theoretical range of 1–5. Higher scores indicate higher levels of negative affect. The Cronbach’s α coefficient in the present study was 0.93.

#### Suicidal ideation

Suicidal ideation was evaluated using the Self-Rating Idea of Suicide Scale (SIOSS) by Xia et al.^80^. This questionnaire comprises 26 items, each measured using a 2-point scoring method (“0” indicating no, “1” indicating yes). The scale encompasses four dimensions: optimism (e.g., ‘In my daily life, there are things that interest me’), sleep (e.g., ‘I sleep restlessly and am easily disturbed’), despair (e.g., ‘It seems like there is no hope for my future’) and dissimulation (e.g., ‘Sometimes I tell lies’). Participants with a dissimulation factor score of 4 or higher were excluded. According to Xia et al.^80^, suicidal ideation was defined as a total score exceeding 12 on the combined measures of despair, pessimism, and sleep. The suicidal ideation score was operationalized as the mean score across the remaining 21 items (spanning the despair, optimism, and sleep dimensions), resulting in a theoretical range of 0–1. Higher total scores (excluding the factor score of dissimulation) indicate stronger suicidal ideation. The Cronbach’s α coefficient in the present study was 0.87.

#### Rejection sensitivity

Rejection sensitivity was evaluated using the Tendency to Expect Rejection Scale (TERS) developed by Jobe^81^. We followed the guidelines for scale translation and cross-cultural adaptation recommended by Sousa and Rojjanasrirat to systematically translate this scale into Chinese^82^. The scale consists of 18 items, such as "I am sensitive to rejection" and "I care a lot about others’ evaluations of me," each rated on a 5-point Likert scale ranging from 1 (“strongly disagree”) to 5 (“strongly agree”). The final score was determined by calculating the mean score of all items, yielding a theoretical range of 1–5. Higher total scores indicate greater levels of rejection sensitivity. The Cronbach’s α coefficient in the present study was 0.78.

#### Social connectedness

Assessment of Social Connectedness (ASC), adapted from Sedikides et al.^83^, was used to gauge social connectedness. We followed the guidelines for scale translation and cross-cultural adaptation recommended by Sousa and Rojjanasrirat to systematically translate this scale into Chinese^82^. It included four items: (1) Right now, I feel connected to loved ones; (2) Right now, I feel protected; (3) Right now, I feel loved; and (4) Right now, I feel I can trust others. Responses were recorded on a 7-point Likert scale, ranging from 1 (“strongly disagree”) to 7 (“strongly agree”). The final score was operationalized by calculating the mean score of all items, resulting in a theoretical range of 1–7. Higher scores indicate a greater sense of social connectedness. The Cronbach’s α coefficient in the present study was 0.91.

## Results

Data analysis for this study was conducted using IBM SPSS Statistics 27.0.

### Common method bias

Harman’s single-factor test was used to assess potential common method bias in our study. There were 10 factors with eigenvalues above 1, and the variance explained by the first common factor was 22.12%, which was below the empirical criterion of 40%. Consequently, no serious common method bias was detected in this study.

### Correlation analysis of main variables

Correlation analyses indicated positive correlations among negative affect, suicidal ideation, and rejection sensitivity. In contrast, social connectedness was negatively correlated with these three variables (see Table 1). The significant correlations among the variables suggest the suitability of the data for subsequent mediation analysis and further hypothesis testing.

**Table 1:** Results of descriptive statistics and correlation analysis.

Variable	*M*	*SD*	1	2	3	4
1 rejection sensitivity	2.76	0.50	–			
2 social connectedness	6.06	1.10	− 0.22**	–		
3 negative affect	1.90	0.69	0.50**	− 0.35**	–	
4 suicidal ideation	0.10	0.15	0.36**	− 0.53**	0.43**	–

All numerical values are reported to two decimal places; *p < 0.05, **p < 0.01, ***p < 0.001.

### Mediation effect test

Previous studies have shown that suicidal ideation, social connectedness, and negative affect vary by age, gender, and only-child status^84–87^. Therefore, we included these three variables as control variables in our study. Additionally, to better understand the high-risk subgroups within our sample, we examined differences in suicidal ideation across these three variables. The specific results were as follows: significant differences were found between only-child and non-only-child groups in suicidal ideation (*t* (752.43) = 2.73, *p* = 0.007, 95% CI [0.16, 0.96]), with the only-child group scoring higher; age showed a significant negative correlation with suicidal ideation (*r* = − 0.14, *p* < 0.001, 95% CI [− 0.19, − 0.08]); while gender differences were not statistically significant (*t* (992.16) =  − 1.81, *p* = 0.07, 95% CI [− 0.73, 0.30]).

Prior to the mediation analyses, the continuous psychological variables were standardized (z-scores). Using rejection sensitivity as the independent variable, social connectedness and negative affect as mediating variables, and suicidal ideation as the dependent variable, the PROCESS macro program (SPSS plugin provided by Hayes, 2013) was employed. To test the hypothesis, Model 6 (chain mediation) was adopted. The chain mediation model was tested using bootstrap sampling with 5,000 resamples and 95% confidence intervals. Results showed a significant positive total effect of rejection sensitivity on suicidal ideation (β = 0.35, 95% CI [0.30, 0.40]), supporting H1. After social connectedness and negative affect were incorporated into the model, the direct effect of rejection sensitivity on suicidal ideation remained significant (β = 0.16, 95% CI [0.11, 0.22]). Specifically, the standardized path coefficient from rejection sensitivity to social connectedness was -0.21, and that from social connectedness to suicidal ideation was -0.42. The path coefficient from rejection sensitivity to negative affect was 0.44, that from negative affect to suicidal ideation was 0.20, and that from social connectedness to negative affect was − 0.24(Table 2).

**Table 2:** Regression analysis of variable relationship in the mediation model.

Outcome variable	Predictor variable	Fit indices			Significance of the coefficient	
		R	R^2^	F	β	t
Social connectedness	Sex	0.25	0.06	18.44	− 0.18	− 3.06
	Age				0.03	2.23
	Only-child				0.05	0.87
	Rejection sensitivity				− 0.21	− 7.45
Negative affect	Sex	0.56	0.31	104.48	0.02	0.44
	Age				− 0.04	− 2.88
	Only-child				− 0.08	− 1.50
	Rejection sensitivity				0.44	17.39
	Social connectedness				− 0.24	− 9.55
Suicidal ideation	Sex	0.61	0.38	115.12	0.04	0.82
	Age				− 0.03	− 2.39
	Only-child				− 0.11	− 2.28
	Rejection sensitivity				0.16	6.06
	Social connectedness				− 0.42	− 16.60
	Negative affect				0.20	7.01

Indirect effects were examined using bias-corrected bootstrap sampling with 5,000 resamples. As shown in Table 3, the total indirect effect was 0.19 (95% CI [0.14, 0.23]), indicating a significant overall indirect effect. This total comprised three pathways: Ind1 (rejection sensitivity → social connectedness → suicidal ideation), effect = 0.09 (95% CI [0.06, 0.12]); Ind2 (rejection sensitivity → negative affect → suicidal ideation), effect = 0.09 (95% CI [0.05, 0.12]); and Ind3 (rejection sensitivity → social connectedness → negative affect → suicidal ideation), effect = 0.01 (95% CI [0.005, 0.02]). The bootstrap 95% confidence intervals for all three indirect pathways excluded zero, indicating significant indirect effects across all pathways. These findings support Hypotheses 2, 3, and 4.

**Table 3:** Bootstrap analysis of the chain mediation model.

Effect	Path	Effect size	SE/BootSE	95% confidence interval	
				**BootLLCI**	**BootULCI**
Total effect		0.35	0.03	0.30	0.40
Direct effect		0.16	0.03	0.11	0.22
Total indirect effect		0.19	0.02	0.14	0.23
Indirect effect	Path 1	0.09	0.02	0.06	0.12
	Path 2	0.09	0.02	0.05	0.12
	Path 3	0.01	0.003	0.005	0.02

Path 1 means rejection sensitivity → social connectedness →suicidal ideation.

Path 2 means rejection sensitivity → negative affect → suicidal ideation.

Path 3 means rejection sensitivity → social connectedness → negative affect → suicidal ideation.

**Fig. 2:**
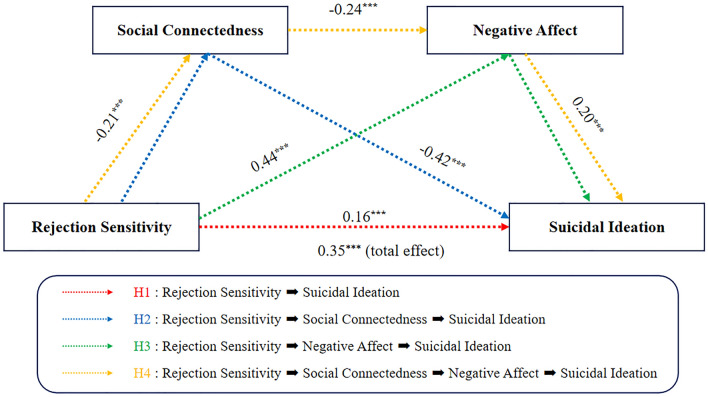
Chain-mediated effects model of social connectedness and negative affect.

According to the above research results, the chain mediation model is shown in Fig. 2

## Discussion

To explore the underlying mechanisms through which rejection sensitivity is associated with suicidal ideation among college students, this study constructed and validated a chained mediation model incorporating social connectedness and negative affect as mediating variables. Overall, the study broadens our understanding of the mechanisms connecting rejection sensitivity to suicidal ideation and provides meaningful implications for prevention and early intervention efforts addressing suicidal ideation among college students.

### Relationship between rejection sensitivity and suicidal ideation

The present study found that rejection sensitivity was significantly and positively associated with suicidal ideation among college students, supporting H1. Furthermore, the direct association between rejection sensitivity and suicidal ideation remained significant after controlling for the mediating effects of social connectedness and negative affect. This finding further corroborates the association between rejection sensitivity and suicidal ideation. Rejection sensitivity has been widely considered a vulnerability factor that contributes to unhealthy psychological states^62,88^. The present study further indicates that excessive anticipation of and reactivity to rejection keep individuals in a state of hypervigilance and defensiveness during interpersonal interactions, and such chronic stress may also increase the risk of suicidal ideation^89,90^. This finding also supports the interpersonal theory of suicide, as this direct effect suggests that individuals with high rejection sensitivity, after experiencing rejection, may perceive themselves as not belonging to a group, thereby experiencing a sense of thwarted belongingness^34,55^. This result is inconsistent with the findings of Brown et al.^33^, who found that rejection sensitivity was not directly associated with suicidal ideation but rather exerted an indirect effect through mediating variables. The reasons for this discrepancy may lie in both the characteristics of the sample and the choice of measurement tools. The present study focused on college students, whose expression of rejection sensitivity may differ fundamentally from that of clinical inpatients. The latter typically present with more complex pathological features, which may result in different patterns of association involving rejection sensitivity^91^. In addition, the measurement tools used differ in their focus: the Adult Rejection Sensitivity Questionnaire (A-RSQ) used by Brown et al. emphasizes individuals’ tendency toward rejection sensitivity within specific interpersonal situations^92^, whereas the Tendency to Expect Rejection Scale (TERS) employed in the present study is context-free and measures individuals’ general cognitive tendency to expect rejection^81^. This difference may further amplify the divergence in findings arising from sample characteristics. Importantly, this discrepancy itself holds value, as it suggests that the effect of rejection sensitivity on suicidal ideation may be conditional—an issue worthy of further investigation in future research.

### Mediating role of social connectedness

The present study found that social connectedness mediates the relationship between rejection sensitivity and suicidal ideation, supporting H2. The negative association between rejection sensitivity and social connectedness reflects the interpersonal interaction patterns of individuals with high rejection sensitivity. They tend to withdraw socially, approach others less frequently during interactions, and perceive the quality of their interactions as more unstable^55,93,94^. This suggests that rejection sensitivity is not only an internal personality trait but also manifests as observable differences in interpersonal behavior. Furthermore, the negative association between social connectedness and suicidal ideation highlights the potential protective role of social connectedness against suicide risk^95,96^. Research has found that when individuals’ social connectedness is weakened, their access to psychological and material resources from others is correspondingly reduced^51^. According to the interpersonal theory of suicide, thwarted belongingness is a key source of suicidal ideation, and a decline in social connectedness is a concrete manifestation of thwarted belongingness^34^. Thus, when individuals lack the external support derived from interacting with others, their ability to cope with adversity diminishes, and the risk of suicidal ideation increases accordingly^96^. This finding is partially consistent with the results of Brown et al.^33^ as both studies emphasize the role of interpersonal factors in the development of suicidal ideation. This suggests that future research on suicidal ideation should adopt an interpersonal perspective, focusing on the state of individuals’ interactions with others and the impact of these interactions on them.

Given that the present study was conducted among Chinese college students, it is important to interpret these findings within the cultural context of collectivism and interpersonal harmony. Chinese students grow up in an environment that emphasizes group belonging and face concern^6^. Living in college dormitories further intensifies interpersonal exposure, making social rejection particularly salient and distressing^7^. Consequently, the pathways identified in this study (rejection sensitivity → reduced social connectedness → negative affect → suicidal ideation) may be especially pronounced in this population. These findings are also consistent with the Escape Theory in the Chinese cultural context, where the pressure to maintain interpersonal harmony and avoid losing face may lower the threshold at which negative affect becomes unbearable.

### Mediating role of negative affect

The present study also found that negative affect mediates the relationship between rejection sensitivity and suicidal ideation, supporting H3. The positive association between rejection sensitivity and negative affect may reflect the tendency of individuals with high rejection sensitivity to experience stronger negative emotional responses. This finding suggests that individuals with high rejection sensitivity, due to their excessive anticipation of and strong reactivity to interpersonal rejection cues, may be more prone to the accumulation of negative affect^97^. At the same time, the emotional dysregulation that often accompanies high rejection sensitivity may make it difficult for their negative affect to be effectively regulated, thereby allowing it to persist^23,63^. The positive association between negative affect and suicidal ideation suggests that individuals with higher levels of negative affect also tend to report higher levels of suicidal ideation. From the perspective of the escape theory, when negative affect reaches a certain level, individuals may initiate a process of cognitive deconstruction in an attempt to escape from distress, which may increase the likelihood of suicidal ideation^66^. This finding is consistent with previous research^60,70,71^. In contrast, previous studies often examined various types of negative affect separately. Those studies found that rejection sensitivity is significantly positively correlated with multiple distinct forms of negative affect and that negative affect such as depression and anxiety each exacerbate suicidal ideation^60,98^. However, research has shown that different forms of negative affect are often highly correlated with one another. In fact, distinct emotions sharing the same negative valence frequently co-occur^99^. Therefore, we measured negative affect as a unified construct, which better reflects real-world conditions.

Beyond measurement considerations, it is also important to acknowledge the potential bidirectionality of the relationship between rejection sensitivity and negative affect. While our theoretical model posits rejection sensitivity as a dispositional vulnerability that precedes and heightens negative affect, individuals with preexisting emotional vulnerabilities (such as high neuroticism or a history of mood disorders) may also be more prone to developing rejection sensitivity^62^. This reciprocal dynamic suggests that rejection sensitivity and negative affect may mutually reinforce each other over time. However, consistent with the conceptualization of rejection sensitivity as a relatively stable cognitive-affective disposition originating from early attachment experiences^18^, our study focused on the directional pathway from rejection sensitivity to negative affect. Future longitudinal research is needed to disentangle the temporal ordering and potential reciprocal effects between these constructs.

### The chain mediation effect of rejection sensitivity on suicidal ideation

Interestingly, the present study identified a significant chain mediation involving social connectedness and negative affect, further illustrating the complex relationship between college students’ rejection sensitivity and suicidal ideation. Considering the overall pathway, these results reveal a process that progresses from cognitive risk to interpersonal disconnection, then to emotional distress, and ultimately to suicidal ideation. Specifically, individuals with high rejection sensitivity tend to show reduced social engagement with others due to anticipating rejection, which may be associated with weakened social connectedness^12^. Weak social connectedness was also associated with reduced access to psychological and material resources from others, potentially rendering individuals more susceptible^51^. When negative affect reaches a level that becomes unbearable, suicide may come to be seen as the only way to escape suffering, and suicidal ideation emerges accordingly^66^. This pathway is consistent with established classical theories^34,66^. The interpersonal theory of suicide emphasizes thwarted belongingness, which corresponds to the weakening of social connectedness in this pathway, while the escape theory explains how negative affect may be linked to suicidal ideation. Thus, our findings provide robust theoretical support for understanding the mechanisms underlying the development and maintenance of suicidal ideation.

Beyond its theoretical contributions, this chain mediation finding also has important practical implications, particularly with respect to the central role of interpersonal processes. Rejection sensitivity, by its very nature, is an interpersonal vulnerability that manifests in social contexts^22^. The finding that reduced social connectedness serves as the initial pathway from rejection sensitivity to negative affect highlights the importance of real-world interactions. Individuals with high rejection sensitivity not only perceive rejection more readily but also actively withdraw from social engagement, thereby depriving themselves of the very connections that could buffer against emotional distress^93^. This suggests that interventions aimed at reducing suicide risk should focus not only on changing cognitive appraisals of rejection but also on facilitating positive interpersonal experiences that can disconfirm expectations of rejection.

### Implications

The findings of this study offer several theoretical and practical implications.

#### Theoretical implications

First, by identifying a chain mediation pathway (rejection sensitivity → social connectedness → negative affect → suicidal ideation), this study extends the existing literature by integrating the Interpersonal Theory of Suicide^34^ and the Escape Theory of Suicide^66^ into a unified framework. This integration suggests that the transition from interpersonal vulnerability to suicidal ideation involves a sequential process. Initial cognitive and behavioral responses to rejection, such as withdrawal and reduced social connectedness, are followed by emotional distress, which is then associated with elevated suicidal ideation. Second, the study provides empirical support for conceptualizing negative affect as a unified construct in the context of interpersonal vulnerability, acknowledging that distinct negative affect often co-occur and may exert cumulative effects on suicide risk. Third, by demonstrating that rejection sensitivity is directly associated with suicidal ideation even after accounting for mediating mechanisms, the findings highlight the importance of considering both direct and indirect pathways in future research.

#### Practical implications

The findings have important implications for suicide prevention and intervention efforts among college students. First, the mediating role of social connectedness suggests that interventions aimed at strengthening social connectedness may be particularly effective in reducing suicide risk. Campus-based programs that facilitate positive peer interactions, promote inclusive social environments, and provide opportunities for students to build meaningful relationships could serve as protective factors. Second, the mediating role of negative affect highlights the importance of emotion regulation skills in suicide prevention. Cognitive behavioral interventions that help students identify, tolerate, and regulate negative affect may disrupt the pathway from rejection sensitivity to suicidal ideation. Third, the chain mediation effect suggests that interventions addressing both interpersonal and emotional components simultaneously may yield greater benefits than targeting either mechanism alone. For example, programs that combine social skills training with emotion regulation strategies could be particularly effective for students high in rejection sensitivity. This is consistent with prior intervention research^100–102^. One study demonstrated that school-based programs integrating social and emotional learning (SEL), which explicitly combine social skills training with emotion regulation strategies, significantly improve students’ social-emotional competencies and reduce emotional distress^102^. Another study concluded that school-based programs strengthening social connectedness are among the most effective suicide prevention strategies^95^. Together, these findings suggest that interventions targeting both interpersonal skills and emotional regulation, as well as those fostering social connectedness, hold promise for reducing suicide risk among college students. Finally, the direct association between rejection sensitivity and suicidal ideation underscores the importance of identifying and supporting students who exhibit high levels of rejection sensitivity, even in the absence of overt emotional distress or social withdrawal. Screening efforts and early intervention programs that target this vulnerable group may help prevent the escalation of suicide risk.

### Limitations and directions for future research

This study has several limitations. First, regarding data collection, the study relied solely on self-report questionnaires. Self-report measures are susceptible to social desirability bias, which may lead participants to underreport sensitive information such as suicidal ideation or overreport socially acceptable behaviors. In addition, the items on the suicidal ideation questionnaire are inherently sensitive, and no specific steps were taken to mitigate potential response bias. Future research could incorporate observational data, interviews, or informant reports to obtain more comprehensive and objective assessments.

Second, the study used a cross-sectional research design, which does not permit causal inference. Although our proposed chain mediation model is grounded in theory, the cross-sectional nature of the data means that we cannot establish the temporal ordering of the variables or rule out alternative directional pathways. For instance, it is possible that negative affect may increase rejection sensitivity over time, or that low social connectedness may precede and exacerbate rejection sensitivity. Future studies should adopt cross-lagged panel designs with at least two or three time points to better establish the temporal ordering among rejection sensitivity, social connectedness, negative affect, and suicidal ideation. Such designs would allow researchers to determine whether changes in rejection sensitivity precede subsequent changes in social connectedness and negative affect, and whether these temporal sequences predict later suicidal ideation, thereby providing stronger evidence for the directional pathways proposed in our model.

Third, regarding the measurement of key constructs, negative affect was assessed as a general dimension rather than as specific emotions such as depression or anxiety. While this approach is supported by evidence that distinct negative affect frequently co-occur and share common underlying mechanisms^99^, it does not allow us to determine whether specific emotions exert differential effects on suicidal ideation. Future research could differentiate between specific forms of negative affect to provide more precise evidence for this relationship and inform targeted intervention strategies. Additionally, since both rejection sensitivity and social connectedness are interpersonal variables, future research could expand to include variables from other domains, such as mental health literacy or life values, to offer a more comprehensive understanding of the mechanisms linking interpersonal vulnerability to suicidal ideation.

Fourth, the mean score of suicidal ideation in our sample was relatively low, indicating that most participants reported minimal suicidal thoughts. This restricted range may limit the generalizability of our findings to clinical populations with elevated suicide risk. Future studies should replicate these findings in samples with greater variability in suicidal ideation, including clinical or help-seeking populations.

Fifth, although prior research has suggested that female college students may be more vulnerable to suicidal ideation^103^, we did not find a significant gender effect in our sample. This may be due to the relatively low mean level of suicidal ideation in our sample, which may have attenuated gender differences. Alternatively, it is possible that the mechanisms linking rejection sensitivity to suicidal ideation operate similarly across genders in nonclinical college populations. Future research with larger samples and greater variability in suicidal ideation is needed to clarify potential gender differences.

Sixth, the present study did not include measures of depressive symptoms or anxiety disorders, which are well-established risk factors for suicidal ideation^104,105^. Given that both depression and anxiety are highly correlated with negative affect and may independently contribute to suicide risk, the absence of these measures limits our ability to determine whether the observed effects are uniquely attributable to negative affect or are confounded by these related conditions. Future research should include clinical assessments of depression and anxiety to more precisely isolate the specific contributions of rejection sensitivity and social connectedness to suicidal ideation.

Seventh, although we included gender and age as control variables in our main analyses, we did not systematically examine subgroup differences (e.g., by grade level, only-child status, or socioeconomic background) to identify which college students are most at risk for suicidal ideation. Future research should adopt more fine-grained analytic strategies to explore these subgroup differences, as such knowledge would inform targeted prevention and intervention efforts.

## Conclusion

In summary, this study explored a chain mediation model linking rejection sensitivity to suicidal ideation among college students. The results indicated that the associations between rejection sensitivity and suicidal ideation were partially accounted for by social connectedness and negative affect. The findings suggest that enhancing social connectedness and promoting emotion regulation skills could be effective targets for interventions aimed at reducing suicidal ideation. Overall, the study broadens our understanding of the mechanisms connecting rejection sensitivity to suicidal ideation and provides meaningful implications for the prevention and early intervention of suicidal ideation among college students.

## Data Availability

The datasets used and analysed during the current study are available from the corresponding author on reasonable request.
